# Spontaneous bidirectional ordering of CH_3_NH_3_^+^ in lead iodide perovskites at room temperature: The origins of the tetragonal phase

**DOI:** 10.1038/srep24443

**Published:** 2016-04-15

**Authors:** Ioannis Deretzis, Bruno N. Di Mauro, Alessandra Alberti, Giovanna Pellegrino, Emanuele Smecca, Antonino La Magna

**Affiliations:** 1 CNR-IMM, Z.I. VIII strada 5, 95121 Catania, Italy; 2 Distretto Tecnologico Micro e Nano Sistemi S.c.a.r.l., Z.I. VIII strada 5, 95121 Catania, Italy

## Abstract

CH_3_NH_3_PbI_3_ is a hybrid organic-inorganic material with a perovskite structure and a temperature-dependent polymorphism whose origins are still unclear. Here we perform *ab initio* molecular dynamics simulations in order to investigate the structural properties and atom dynamics of CH_3_NH_3_PbI_3_ at room temperature. Starting from different initial configurations, we find that a single-crystalline system undergoes a spontaneous ordering process which brings the 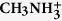 ions to alternately point towards the center of two out of the six faces of the cubic 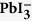 framework, i.e. towards the 〈100〉 and 〈010〉 directions. This bidirectional ordering gives rise to a preferential distortion of the inorganic lattice on the **a**-**b** plane, shaping the observed tetragonal symmetry of the system. The process requires tens of picoseconds for CH_3_NH_3_PbI_3_ supercells with just eight 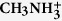 ions.

Methylammonium lead iodide (CH_3_NH_3_PbI_3_) is an excellent material for light harvesting^1^,^2^,^3^,^4^ and photonic^5^ applications, with a solar cell efficiency that is nowadays comparable to that of silicon^6^. Current research is mainly dedicated to the optimization of growth and processing methods in order to enhance device performance and overcome stability issues^7^,^8^. Notwithstanding this rapid technological growth, many questions regarding the fundamental properties of this material are still open. One of the most important issues that needs further understanding is the origin of polymorphism: CH_3_NH_3_PbI_3_ belongs to a group of ABX_3_ perovskite crystals (A, B: cations, X: anion) that typically have a cubic structure. Unlikely, the cubic symmetry is only verified for temperatures *T* > 327 °K. At lower temperatures, the system crystallizes with either an orthorombic (*T* < 162° − 165 °K) or a tetragonal (165° < *T* < 327 °K) symmetry^9^,^10^. Moreover, reversible phase transitions can occur by simply varying the external temperature^9^,^10^. We note that the main distinctive characteristic of the three CH_3_NH_3_PbI_3_ phases is the mean bond-length along the three axes of the 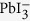 inorganic framework. With this respect, only the cubic phase is isotropic, whereas the tetragonal phase is partially isotropic (it has two out of three equivalent directions) and the orthorombic phase is totally anisotropic.

The temperature-dependent polymorphism of CH_3_NH_3_PbI_3_ brings to attention the unknown origins of phase stability in this material. Previous studies have correlated this issue with the presence of the 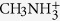 cation, the respective distortion that this induces to the inorganic cage and its degree of orientational disorder^10^,^11^. Indeed, it has been demonstrated from theoretical calculations that the lack of spherical symmetry in the 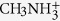 ion affects also the surrounding inorganic cage (through 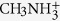-I^−^ interactions) and consequently, the bond-lengths between inorganic ions^12^,^13^. Although such approach could partially explain the orthorobmic (ordered 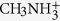) and cubic (disordered 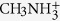) phases, difficulty to correlate the tetragonal phase to a scheme of “middle” disorder appears. Within this context, both experimental and theoretical studies have been performed, with experimental results often increasing the debate on the argument^10^,^11^,^14^,^15^,^16^,^17^. It also remains unclear whether scattering interactions during measurements can impact on the rotation of the 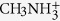 ions, as the relative rotation barriers are low^18^. On the other hand, static density functional theory (DFT) calculations usually end up predicting quasi-isoenergetic metastable configurations, regardless of the 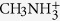 orientation^12^.

The intrinsic difficulty in defining the exact structural properties of CH_3_NH_3_PbI_3_ crystals makes necessary the implementation of advanced computational techniques that are able to fully account for the dynamics of the 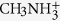 ions. This can be achieved through *ab initio* molecular dynamics simulations^19^. In addition to previous studies^20^,^21^,^22^,^23^,^24^,^25^, our work aims at understanding the origins of the tetragonal phase by means of extended simulation times (up to 65 *ps*) and different initial configurations. Our results indicate that through combined organic-inorganic interactions at room temperature, there exists a spontaneous ordering mechanism that brings the ammonium of the 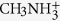 ions to point towards two out of the six faces of the 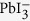 inorganic framework, i.e. towards the 〈100〉 and 〈010〉 directions. The accompanying preferential distortion of the inorganic lattice shapes the tetragonal symmetry of the system.

## Results

**Figure 1:**
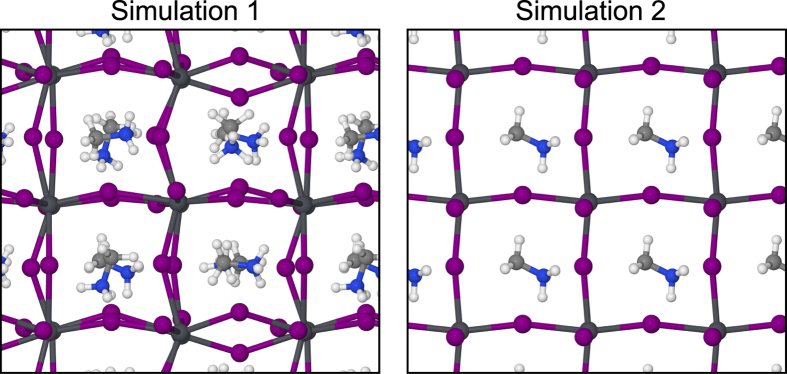
Initial configurations for the two Car-Parrinello simulations, based on (2 × 2 × 2) cubic supercells of CH_3_NH_3_PbI_3_.

**Figure 2:**
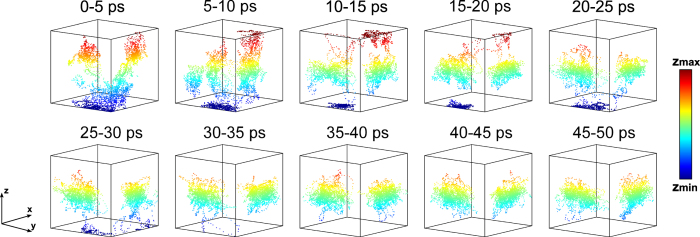
Orientation of the 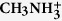 ions (considering the -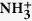 part) projected on the cubic 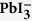 inorganic framework for every 5 *ps* of simulation time. The system undergoes a spontaneous ordering process which brings the 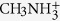 ions to point towards two out of the six faces of the cubic inorganic cage. The colorscale is relative to the *z* coordinate.

**Figure 3:**
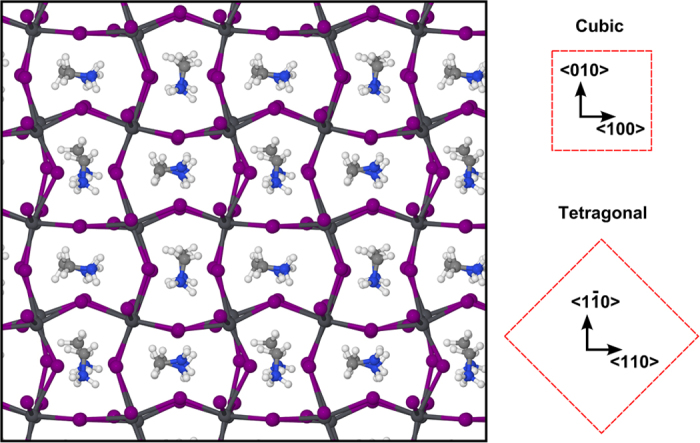
Snapshot of the stable CH_3_NH_3_PbI_3_ configuration at the end of the spontaneous ordering process of the 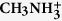 ions. The 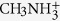 ions point towards the 〈100〉 and 〈010〉 directions of the cubic 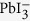 framework, which correspond to the 〈110〉 and 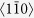 directions of the tetragonal phase.

**Figure 4:**
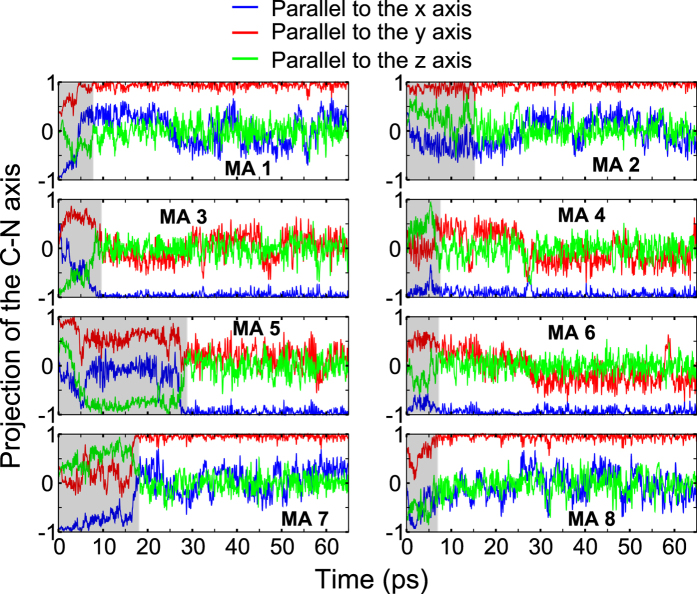
Projection of the C-N axis on the *x*, *y* and *z* directions of the cubic 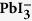 framework for each methylammonium (MA) ion within the simulation cell. Areas highlighted with gray indicate the approximate time needed for the ordering of each MA ion. Projection values with an opposite sign indicate opposite C-N polarities.

As confirmed by numerous experimental studies^9^,^10^, CH_3_NH_3_PbI_3_ crystallizes with a tetragonal symmetry at room temperature. This aspect usually impacts on the choice of the supercell for *ab initio* molecular dynamics calculations. However, the subtle point to take into account here is that the tetragonal phase should be regarded as a *result* of particular atom dynamics and not as an intrinsic property of the crystal structure of the material, which otherwise should be cubic. In this study we consider the cubic symmetry as a starting point in order to understand why the atom kinetics lead to the tetragonal phase. We begin with our first simulation, where the initial position of each methylammonium ion is unique with respect to the others and does not point to any high symmetry direction (see Fig. 1). Figure 2 shows the alignment of the 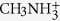 ions projected on the cubic inorganic cage for every 5 *ps* of simulation time up to *t* = 50 *ps*. The first period of the simulation (~0–28 ps) the perovskite structure undergoes an extremely slow reorganization process, where two types of 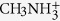 rotations take place: 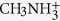 molecular rotations within the inorganic cage (they may occur after few or tens of *ps*) and rotations around the 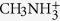 C-N axis (with a higher frequency). In both cases, the rotational activity is discontinuous, rather than periodic. At the end of this period all 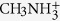 ions gradually rearrange, with their -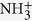 part alternately pointing towards the center of two out of the six faces of the cubic 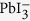 framework. This spontaneous ordering process gives rise to a well-defined crystal structure, which is shown in Fig. 3. For the rest of the simulation time (~28–65 *ps*) such structural configuration remains unaltered. The only rotational movement here is around the C-N axis, which does not alter the direction of the 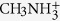 ions. The 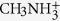 kinetic behavior for the entire simulation time can be seen in Fig. 4, where each C-N vector is projected on the three crystallographic axes of the cubic framework. Here, rotational events that change the 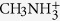 orientation are characterized by steep changes in the projection values along the three crystallographic axes. Usually, such events have a duration of few picoseconds, while the times of metastable configurations may be as long as tens of picoseconds (see methylammonium n. 5 in Fig. 4). After ~28 *ps*, the structure stabilizes in the bidirectional scheme discussed above, and only vibrational effects can be observed for the 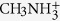 cations.

Our first calculation clearly shows a spontaneous disorder-to-order process with a well-defined final structure. The question that arises though is if such behavior is generic or particular to the initial conditions of such simulation. To this end, we performed a second simulation with entirely different initial conditions, having all 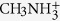 ions pointing towards the 〈120〉 cubic direction (see Fig. 1). Results (see Supplementary Information) confirmed the slow alignment of the 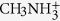 ions towards two out of the six faces of the cubic 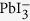 framework after ~41 ps. In this case a transformation from one ordered configuration to a different ordered configuration took place. Interestingly, the 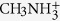 ions ended up being parallel to the *y* and *z* axes of our simulation cell, rather than the *x* and *y* ones (as in our previous calculation). Therefore, the absolute ordering of the 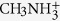 ions inside a perovskite crystal depends on their initial configuration.

Our kinetic results bring to attention the issue of relaxation time for a CH_3_NH_3_PbI_3_ crystal, defined as the time needed for the transition from an out-of-structural-equilibrium state towards structural equilibrium. Our calculations show that this time is of the order of tens of picoseconds (at room temperature) for a simulation cell having only eight 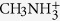 ions. Considering the gradual and collaborative character of the ordering process as well as the actual sizes of single-crystalline grains^2^, it becomes clear that an estimation of relaxation times in real crystals should be definitely orders of magnitude higher than the picosecond-scale accessible to our Car-Parrinello simulations. This aspect also points out that there may be a relationship between time-dependent behaviors often observed in CH_3_NH_3_PbI_3_ crystals and the relaxation mechanisms of the 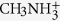 ions (e.g. the conductance hysteresis^26^). Further research is necessary in order to clarify this issue. Similarly, we also stress that simulations with bigger supercells than the ones used in this work are expected to require more time prior to reaching the equilibrium state.

**Figure 5:**
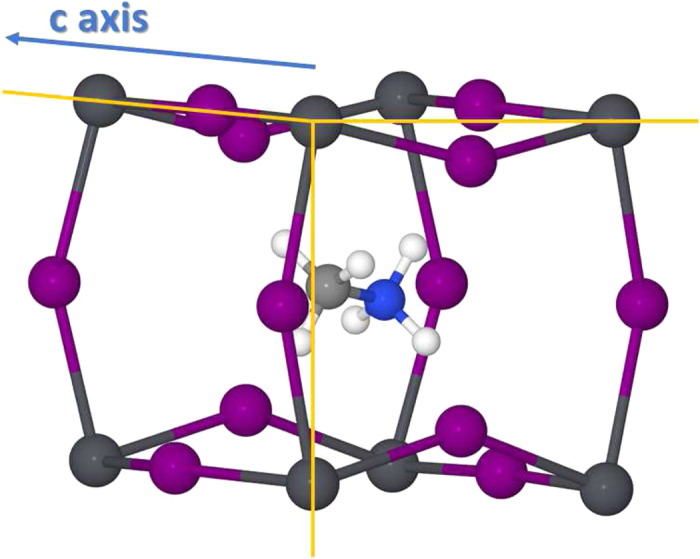
View of a single 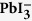 inorganic cage after structural and cell relaxation, showing the preferential distortion of the Pb-I-Pb bonds along two out of the three crystallographic directions.

In order to explore a possible relationship between the spontaneous bidirectional alignment of 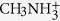 and the tetragonal phase, we performed *variable-cell* DFT calculations, maintaining the same calculation parameters as in our Car-Parrinello simulations and considering the final structural configuration of our molecular dynamics run as the input for the DFT calculation. Upon relaxation of both atomic positions and cell parameters, we indeed found that the lengths of the 〈100〉 and 〈010〉 lattice vectors were smaller than the 〈001〉 vector by ~2.4%, due to a preferential distortion of the inorganic lattice at the 〈100〉-〈010〉 plane from enhanced 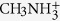-I^−^ interactions (Fig. 5). This aspect is in qualitative agreement with what is expected for the tetragonal *I*4/*mcm* phase at room temperature^11^ and defines the **a**-**b** plane as the one formed by the bidirectional orientation of the 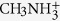 ions. Consequently, the **c** axis has no parallel methylammonium. Hence, in terms of the tetragonal symmetry, the 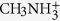 should point towards the 〈110〉 and 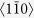 directions, which now correspond to the centers of the 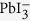 framework (see Fig. 3).

**Figure 6:**
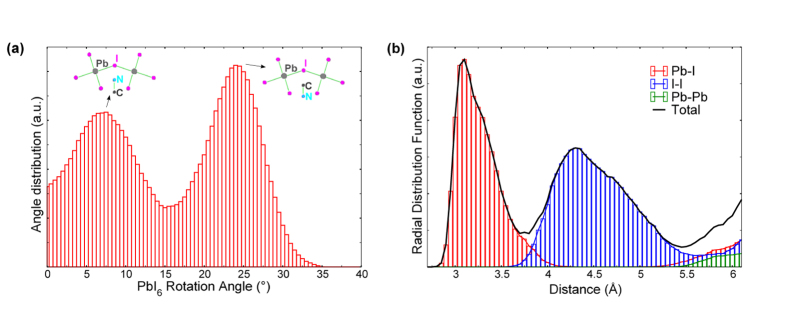
(**a**) Rotational bond-angle distribution for the PbI_6_ octahedra along the *x*-*y* plane in CH_3_NH_3_PbI_3_. The two peaks in the distribution correspond to interactions of the I ions with either the -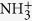 or the -CH_3_ part of the methylammonium ions. (**b**) Radial distribution function for the inorganic part of CH_3_NH_3_PbI_3_, showing the Pb-I, I-I and Pb-Pb contributions.

A further marker of the tetragonal phase is the tilting angle between successive octahedra on the **a**-**b** plane of the system, as defined by Kawamura *et al.*^11^. This rotation of the PbI_6_ octahedra is also clear from the results of our Car-Parrinello simulations, as seen in Fig. 3. An interesting aspect that emerges from the statistical calculation of the distribution for these angles is the presence of two peaks at 7.5° and 24° (see Fig. 6(a)). The origin of this double-peak can be traced at the interaction between the 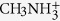 and I^−^ ions, which is enhanced when the -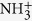 part points towards I atoms. This characteristic leads to a shortening of the 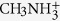-I^−^ distance and a consequent reduction of the respective tilting angle between the two nearby octahedra (7.5° peak). Similarly, the 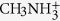-I^−^ interaction is weak when the -CH_3_ part of the 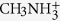 ion points towards an I atom and the respective tilting angle maintains higher values (24° peak). Finally, an important kinetic issue is the bond length distribution of the inorganic lattice upon structural stabilization. Figure 6(b) shows the radial distribution function calculated for the 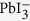 inorganic framework after the ordering of the 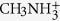 ions. We note that although the first peak corresponding to the Pb-I bond has a maximum at ~3.15 Å, its tail is extended up to ~4 Å, indicating a wide bond-length distribution for the inorganic cage. This is characteristic of the soft mechanical character of the material, and originates from the competitive behavior between 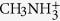 and Pb^2+^ cations, when more than one methylammonium is bound to the same I^−^ anion during thermal movement. When focusing on a single PbI_6_ octahedron (see the Supplementary Information), the Pb-I bonds are highly unbalanced along two out of the three crystallographic directions, whereas they appear almost symmetric for the third crystallographic direction (which corresponds to the **c** axis of the system).

We would finally like to point out that the experimental scenario should be significantly more complex, as CH_3_NH_3_PbI_3_ usually has a polycrystalline and non-textured form^8^. Moreover, lattice imperfections are expected to act as symmetry breaking centers that should induce disorder and obstacle the ordering process discussed above. Similarly, simulations that go beyond the spatial and temporal limitations of *ab initio* molecular dynamics could be necessary in order to study the emergence of a rotational glassy system, as it was recently reported by Fabini *et al.*^27^, or the tetragonal-orthorhombic phase transition that takes place at lower temperatures.

## Discussion

Our calculations show that the tetragonal phase in a defect-free single-crystalline CH_3_NH_3_PbI_3_ system should have an ordered and well-defined structure, where the 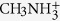 ions point towards the center of two out of the six faces of the cubic 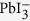 framework. Moreover, the spontaneous alignment of 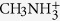 ions indicates that if the system undergoes a momentary structural perturbation (e.g. due to temperature annealing, irradiation or conduction of current), the structural equilibrium should be restored as soon as the perturbative event ends. This last aspect explains the reversibility of the tetragonal-cubic phase transition in this material simply by adjusting the external temperature. Further implications of such ordering process may regard the degree of ferroelectricity^28^,^29^,^30^ as well as time-dependent aspects like the conductance hysteresis and the photovoltaic operation.

To conclude, in this work we have employed Car-Parrinello molecular dynamics simulations in order to study the structural properties and atom dynamics of CH_3_NH_3_PbI_3_ crystals at room temperature. Our calculations have evidenced a slow and spontaneous ordering process of 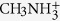 ions which ends up in a bidirectional ordering towards the center of two out of the six faces of the inorganic 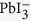 framework. The confined movement of the methylammonium ions on the **a**-**b** plane shapes the tetragonal symmetry of the system. Moreover, the spontaneous character of such process explains the reversible phase transitions upon temperature alterations. Our results are relevant for the better understanding of phase stability and the temperature-dependent behavior of CH_3_NH_3_PbI_3_ crystals.

## Methods

The study was based on Car-Parrinello molecular dynamics^19^ calculations as implemented in the Quantum Espresso software suite^31^. The wave functions and the electronic density were expanded on a plane-wave basis set with a cutoff of 35 Ry and 280 Ry, respectively. The Perdew-Burke-Ezernhof implementation^32^ of the generalized gradient approximation was used for the description of the exchange-correlation functional, along with ultrasoft pseudopotentials^33^. The integration of the equations of motion took place with a time step *dt* = 4 a.u. (i.e. *dt* ≈ 0.1 *fs*), which is small enough to minimize both round-off and truncation errors of the Verlet algorithm^34^. The target temperature for all simulations was set to *T* = 295 °K by means of a Nose thermostat^35^. No constrictions were imposed to the movement of atoms. A 100 a.u. value was assigned for the effective electronic mass whereas ionic masses were set to real values. All simulation cells were based on 2 × 2 × 2 cubic supercells (96 atoms) with a lattice parameter |**a**| = 6.279 Å assigned from X-ray diffraction data^9^. We point out that the 2 × 2 × 2 cubic supercell is double the size and contains the periodicity of the tetragonal unit cell (*I*4/*mcm* space group). Finally, in order to evaluate the effect of structural dynamics on the lattice parameters of the perovskite system, variable-cell density functional theory calculations were performed, maintaining the same computational setup as in the Car-Parrinello calculations and using a 4 × 4 × 4 Monkhorst-Pack grid^36^ for the sampling of the Brillouin zone.

## Additional Information

**How to cite this article**: Deretzis, I. *et al.* Spontaneous bidirectional ordering of CH_3_NH_3_^+^ in lead iodide perovskites at room temperature: The origins of the tetragonal phase. *Sci. Rep.* **6**, 24443; doi: 10.1038/srep24443 (2016).

## Supplementary Material

Supplementary Information
