# Supplementary material for: Spontaneous bidirectional ordering of CH3NH3+ in lead iodide perovskites at room temperature: The origins of the tetragonal phase
Source: Sci Rep. 2016 Apr 15;6:24443. doi: 10.1038/srep24443 (PMC4832208; doi:10.1038/srep24443)
Supplement: Supplementary Information [file srep24443-s1.pdf]

<u>*Supplementary information for:*</u>

Spontaneous bidirectional ordering of $CH_3NH_3^+$ in lead iodide perovskites at room temperature: The origins of the tetragonal phase

Ioannis Deretzis,[1] Bruno N. Di Mauro,[2] Alessandra Alberti,[1] Giovanna Pellegrino,[1] Emanuele Smecca,[1] and Antonino La Magna[1]

[1] CNR-IMM, Z.I. VIII strada 5, 95121 Catania, Italy

[2] Distretto Tecnologico Micro e Nano Sistemi S.c.a.r.l., Z.I. VIII strada 5, 95121 Catania, Italy

1. Results of the second *ab initio* molecular dynamics simulation

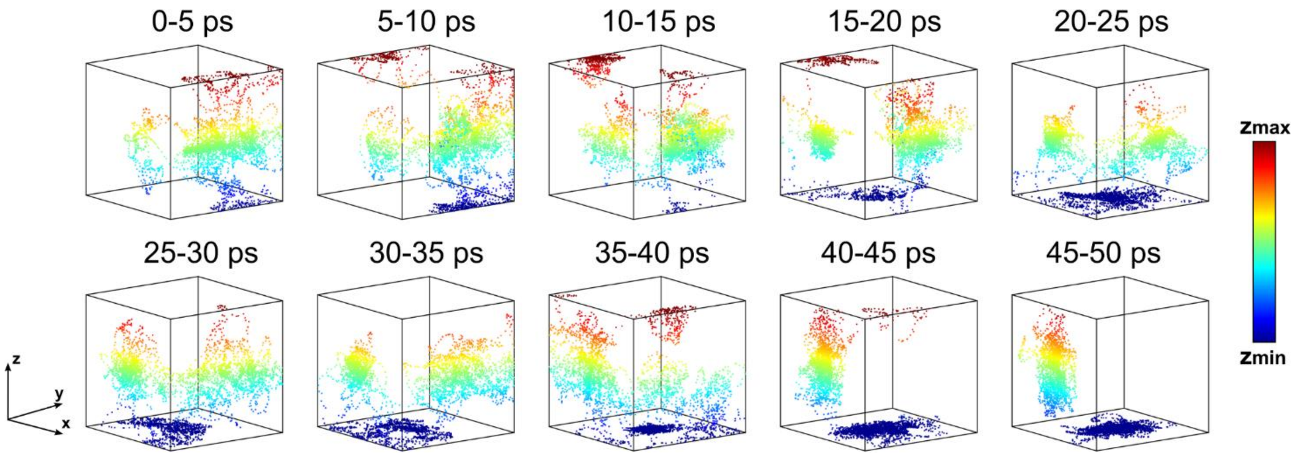

Figure S1. Orientation of the $CH_3NH_3^+$ ions (considering the $–NH_3^+$ part) projected on the cubic PbI_3^- inorganic framework for every 5 *ps* of simulation time. The system undergoes a spontaneous ordering process, which brings the $CH_3NH_3^+$ ions to point towards two out of the six faces of the cubic inorganic cage. The colorscale is relative to the z coordinate.

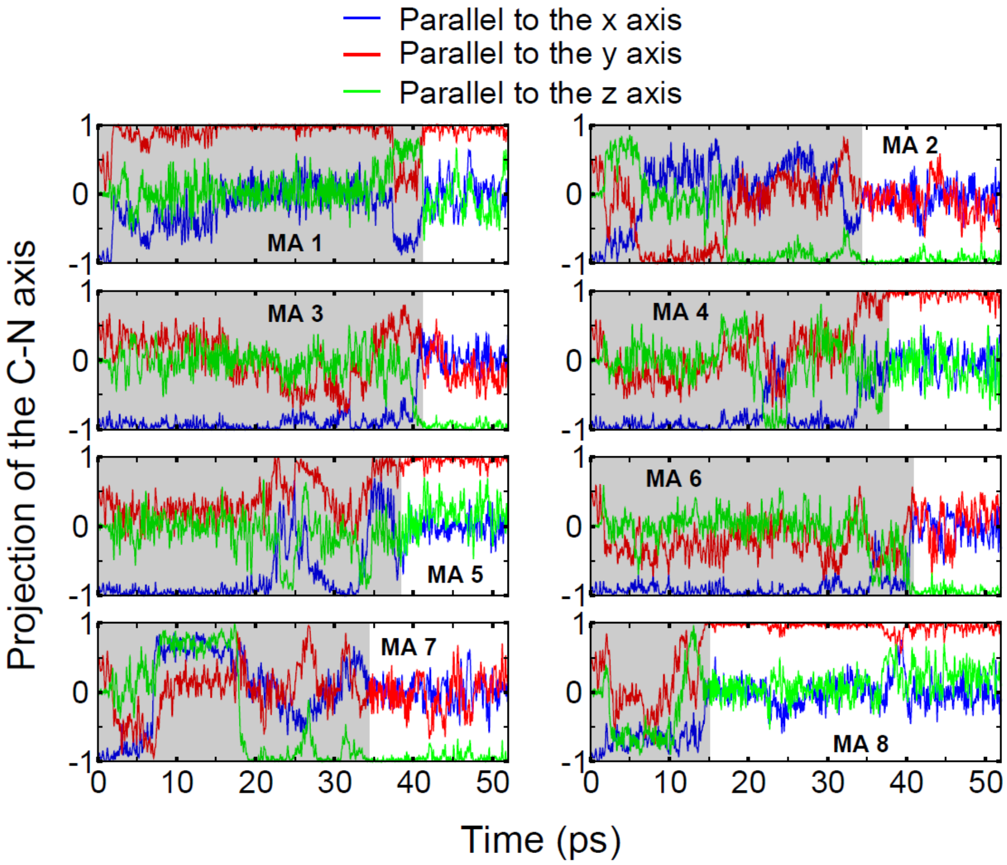

Figure S2. Projection of the C-N axis on the *x*, *y* and *z* directions of the cubic PbI_3^- framework for each methyl-ammonium (MA) ion within the simulation cell. Areas highlighted with gray indicate the approximate time needed for the ordering of each MA ion. Projection values with an opposite sign indicate opposite C-N polarities.

2. Pb-I bond-length distributions for a single PbI_6 octahedron

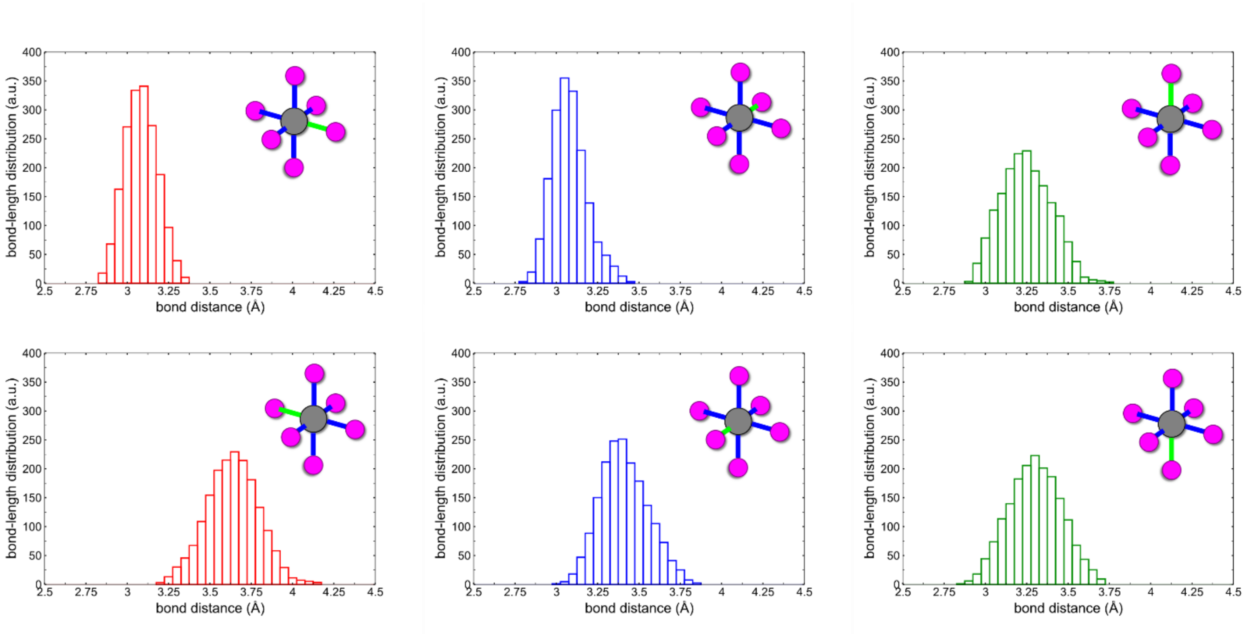

Figure S3. Pb-I bond-length distributions for a single PbI_6 octahedron after the bidirectional ordering of the $CH_3NH_3^+$ ions. Pb-I bonds are highly unbalanced along two out of the three crystallographic directions, whereas they appear almost symmetric for the third crystallographic direction (which corresponds to the **c** axis of the system).
